# Identification and Characterization of MicroRNAs in Normal Equine Tissues by Next Generation Sequencing

**DOI:** 10.1371/journal.pone.0093662

**Published:** 2014-04-02

**Authors:** Myung-Chul Kim, Seung-Woo Lee, Doug-Young Ryu, Feng-Ji Cui, Jong Bhak, Yongbaek Kim

**Affiliations:** 1 Laboratory of Clinical Pathology, College of Veterinary Medicine, Seoul National University, Seoul, Republic of Korea; 2 Laboratory of Environmental Health, College of Veterinary Medicine, Seoul National University, Seoul, Republic of Korea; 3 Research Institute for Veterinary Science, College of Veterinary Medicine, Seoul National University, Seoul, Republic of Korea; 4 Theragen Bio Institute, Suwon-city, Gyeonggi-do, Republic of Korea; SAINT LOUIS UNIVERSITY, United States of America

## Abstract

The role of microRNAs (miRNAs) as a post-transcriptional gene regulator has been elucidated in a broad range of organisms including domestic animals. Characterization of miRNAs in normal tissues is an important step to investigate the functions of miRNAs in various physiological and pathological conditions. Using Illumina Next Generation Sequencing (NGS) technology, we identified a total of 292 known and 329 novel miRNAs in normal horse tissues including skeletal muscle, colon and liver. Distinct sets of miRNAs were differentially expressed in a tissue-specific manner. The miRNA genes were distributed across all the chromosomes except chromosomes 29 and 31 in the horse reference genome. In some chromosomes, multiple miRNAs were clustered and considered to be polycistronic transcript. A base composition analysis showed that equine miRNAs had a higher frequency of A+U than G+C. Furthermore, U tended to be more frequent at the 5′ end of miRNA sequences. This is the first experimental study that identifies and characterizes the global miRNA expression profile in normal horse tissues. The present study enriches the horse miRNA database and provides useful information for further research dissecting biological functions of miRNAs in horse.

## Introduction

MicroRNAs (miRNAs) play a crucial role in various physiological and pathological conditions in a broad range of organisms from viruses to animals [1]–[2]. The short non-coding RNAs regulate the expression of thousands of genes by partial or complementary binding to target mRNAs, resulting in translational inhibition and/or degradation of the target mRNAs [3]–[5]. The mature miRNAs are generated from serial stepwise processing [6]. The enzyme Drosha cleaves the single stranded primary miRNA transcripts(pri-miRNAs) to produce a precursor stem-loop secondary structure(pre-miRNAs) [7]. In turn, the pre-miRNAs are cleaved by the enzyme Dicer to produce mature miRNA duplexes [8]. Only one of the two strands acts as a regulatory RNA associated with RNA-induced silencing complex (RISC) [9].

The importance of miRNAs has been recently recognized in veterinary medicine. There is increasing interest in canine miRNAs because of their clinical relevance for human breast cancers [10]–[11]. Down-regulation of miR-130 and miR-30 in canine heart plays an important role in the pathogenesis of chronic atrial fibrillation [12]. Distinct sets of miRNAs including miR-122, miR-193b, and miR-483 were reported to be involved in the development of type-2 diabetes mellitus in cats [13]. A large number of miRNAs have been identified in normal tissues of swine and bovine species [14]–[17]. However, information about equine miRNAs and their role in clinical conditions is scarce. Recently, *in silico* analytical methodology was applied to identify and characterize mature 354 equine miRNAs [18]. A study revealed 82 new miRNAs in equine sperms, suggesting the role of miRNAs in the regulation of sperm function, fertility and reproduction [19]. Barrey et al. [20] described the expression profile of muscular miRNAs in healthy and myopathic horses using a direct cloning technology.

Forward genetics, bioinformatics and direct cloning have been utilized to study miRNAs [21]. Recently, next generation sequencing (NGS) technology has emerged as a major tool to scrutinize small RNAs including miRNAs [22]. An important feature of the NGS is parallel sequencing of clonally amplified or single DNA molecules that are spatially separated in a flow cell [23]. Due to its ability to generate millions of reads with determined lengths, NGS greatly improves the capacity to identify a large number of novel miRNAs on a genomic scale [24].

Domestic horse, *Equus caballus*, has been a crucial part of human civilization [25]–[26]. It is economically important throughout the world and has been used for transportation and entertainment. Moreover, horse is a medically valuable animal model because the species shares over 90 hereditary conditions with human disorders [27]–[28] as well as many medical conditions such as allergies and osteoarthritis [29]–[30]. Furthermore, horse can be a valuable model organism for studying biomechanics and exercise physiology [31]. The present study was performed to characterize miRNAs in normal horse tissues by using Illumina high-throughput NGS technology. Our study identifies a list of known miRNAs as well as candidates for novel miRNAs in horse tissues. Additionally, characteristics of horse microRNAs including expression patterns in major tissues, sequence composition and chromosomal distribution were determined. Our study provides background data and information to facilitate research on the role of miRNAs in the pathogenesis of various conditions in horses.

## Results

### Library construction of small RNAs from horses

We performed high-throughput short read sequencing of small RNAs from skeletal muscle, liver, and colon tissues from eight Thoroughbred horses. A number of reads, with counts ranging from 11973300 to 21838589, were obtained from the cDNA libraries of small RNAs, of which approximately 100% were of high quality. After eliminating 3′ adapter null, 5′ adapter contaminants, insert null, smaller than 18 nucleotides (nts) and poly A sequences, more than 94% of clean reads in all tissue samples were subjected to further analyses using bioinformatic tools (Table S1). The resultant small RNAs ranged from 18 to 30 nts in length, with the majority having a length of 22 nt.

In general, small RNA libraries generated by NGS is complex in composition because they harbor a large number of degradation fragments derived from coding and noncoding transcripts [32]. To annotate the small RNAs generated by NGS, we performed a BLAST that search of the small RNAs against the equine genome database. Unique small RNAs from muscle, colon, and liver tissues were annotated against the NCBI Genbank and Rfam databases. The annotation results showed that non-coding RNA including exons, introns, repeats, rRNA, scRNA, snRNA, snoRNA, srpRNA, and tRNA accounted for a large percentage of the unique read counts. It also revealed that miRNAs constituted a small portion of the unique read counts but a significant portion of overall read counts (Table S2). More than 70% of unique sequences in all the tissues could not be classified. The sequences of unannotated small RNAs that could be mapped to the equine genome were subject to further analyses to identify novel miRNA candidates.

### Expression profile of miRNAs in major organs

**Figure 1:**
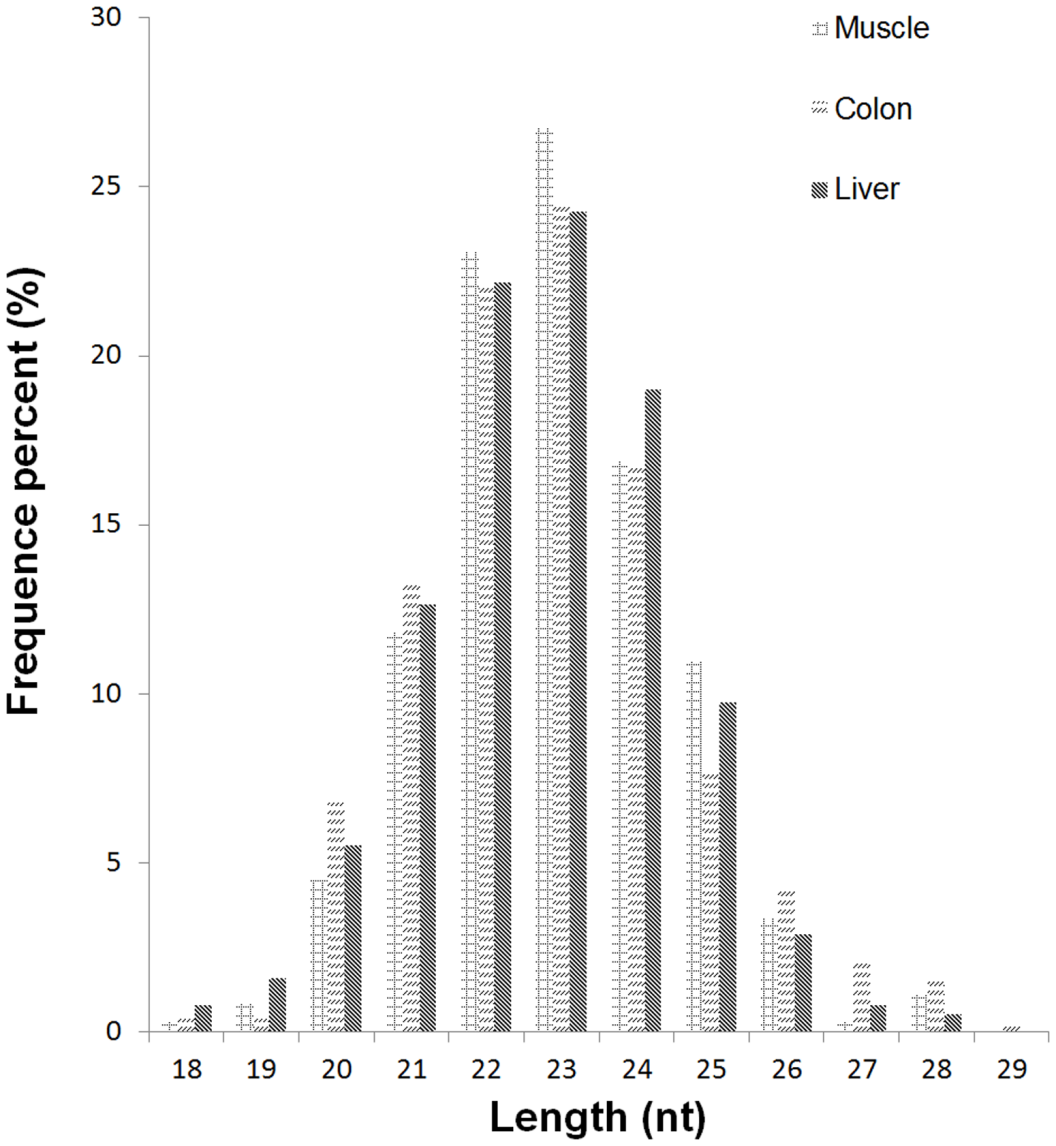
Length distribution and abundance of all miRNAs in horse muscle, colon, and liver tissues. Approximately 83% of all sequences in horse tissues are concentrated in the 20–24 nt range. The most frequent length is 23 nt in all tissues.

**Figure 2:**
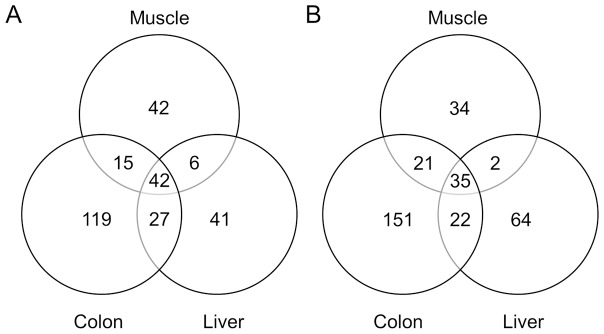
Venn diagram representing the distribution of known and novel miRNAs in horse muscle, colon, and liver tissues. Counts in the Venn diagram are the number of miRNAs identified in each tissue. A total 292 known (A) and 329 novel miRNAs (**B**) are identified in horse tissues including muscle, colon, and liver.

To identify previously reported miRNAs in horse tissues, the small RNAs were submitted for a BLAST search against known equine miRNAs and their precursors in the latest miRBase release version 15.0 [32]. As a result, a total of 201 families including 292 known miRNAs were detected across horse samples (Table S3). The length distribution of known miRNAs was similar in all samples mainly 22–24 nts (Figure 1). The expression profile of known miRNAs in horse tissues was analyzed (Figure 2A). Subsets of tissue-specific miRNAs were identified: 36 miRNAs in muscle samples, 99 miRNAs in colon samples, and 31 miRNAs in liver samples (Table S4).

### Identification of novel equine miRNAs

**Figure 3:**
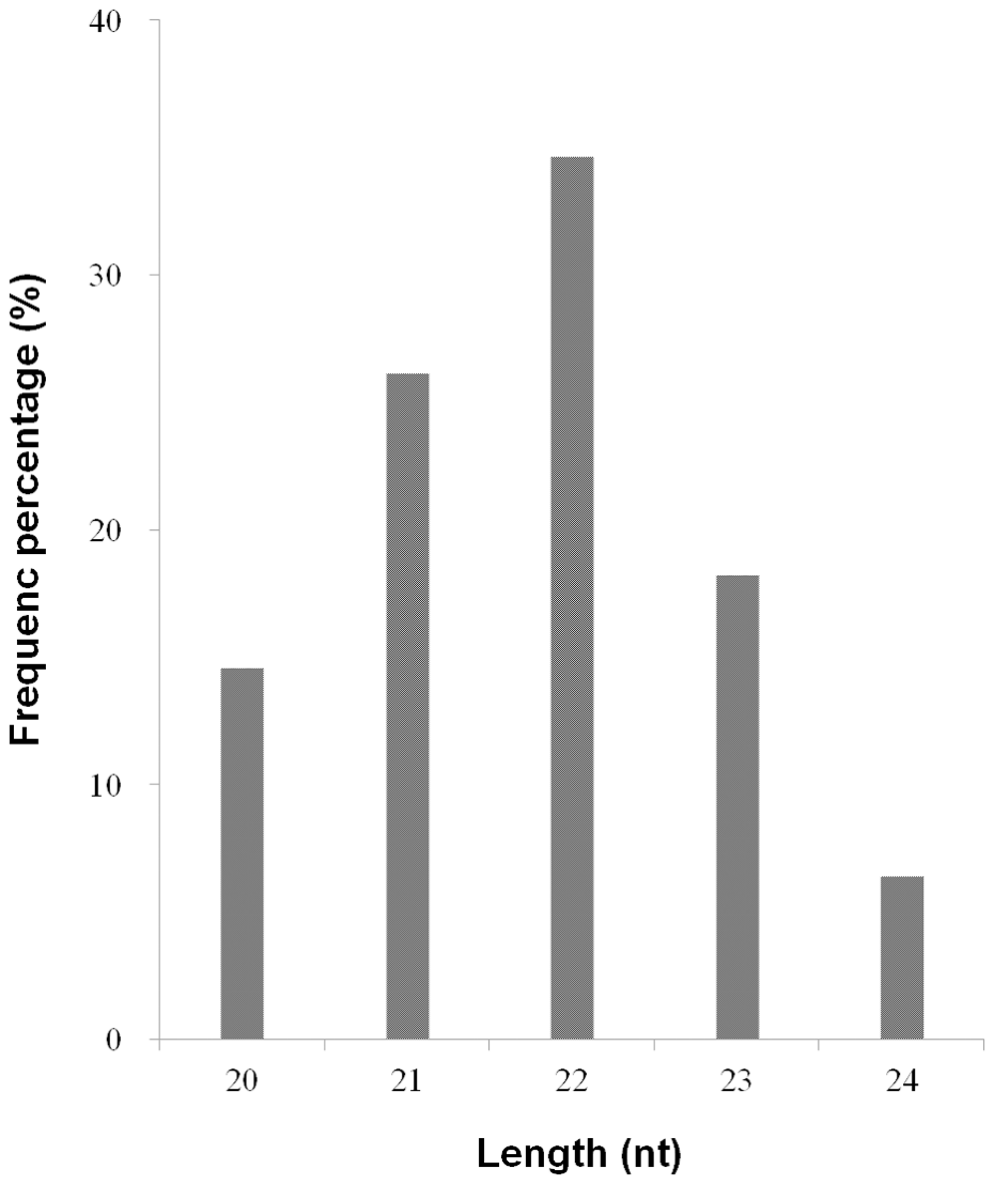
Length distribution of novel miRNAs in horse tissues. miRNA sequences of all lengths are distributed in the 20–24 nt range. The most frequent length is 22 nt (34.65%) in horse miRNAs.

To identify candidates for novel equine miRNAs, a prediction software Mireap (http://sourceforge.net/projects/mireap/) was used to determine secondary structures, the Dicer cleavage sites and the minimum free energy of the unannotated small RNA tags [33]. Based on the analytical criteria, a total of 329 unannotated miRNA sequences were determined as candidates for novel miRNAs in horse (Table S5). The putative novel miRNAs exhibited a concentrated length distribution between 20 nt and 24 nt, with a peak at 21∼22 nt (Figure 3). Expression profile of the novel miRNAs in horse tissues was investigated (Figure 2B). Similar to the known miRNAs, subsets of novel miRNAs were expressed in a tissue-specific manner: 31 novel miRNAs in muscle samples, 123 novel miRNAs in colon samples, 45 novel miRNAs in liver samples (Table S6). Read counts of most novel miRNAs were less than 100 (Data not shown).

### Mapping to genome and miRNA transcriptional units

**Figure 4:**
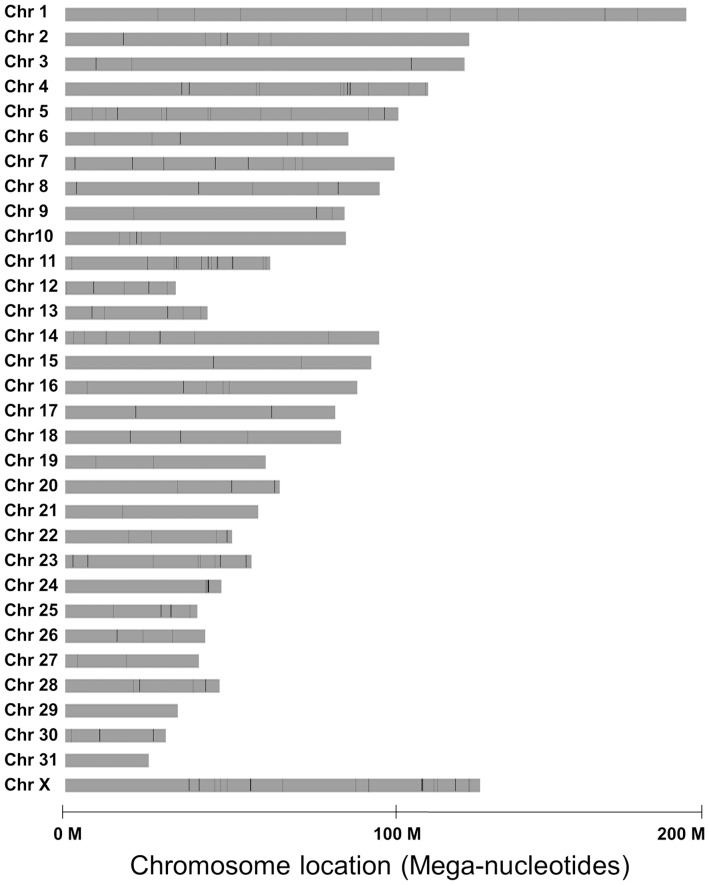
Chromosomal locations of 292 known miRNA genes in horse. Black vertical lines represent the miRNA gene, the depth of color represents the number of miRNA genes in this region. The relative locations of individual miRNAs across the 32 horse chromosomes are shown with the exception of chromosomes 29 and 31.

**Figure 5:**
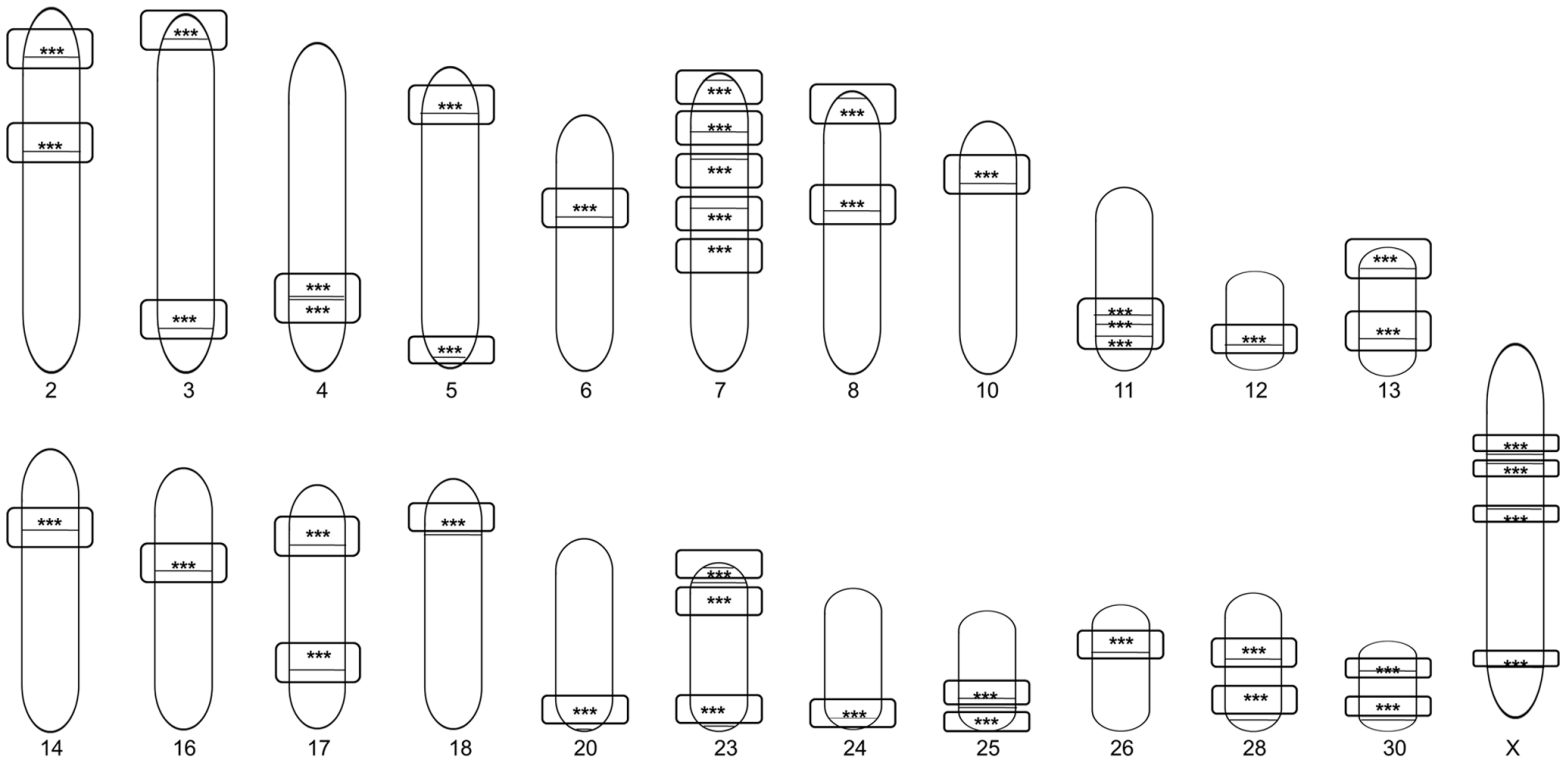
Chromosomal locations of polycistronic miRNAs in horse. Individual black horizontal lines represent polycistronic miRNA transcripts, the stars refer to multiple miRNA genes in this region. The relative locations of individual miRNAs that are considered as a polycistron are within 3

A total of 292 known miRNAs were mapped across the 32 horse chromosomes with the exception of chromosomes 29 and 31 (Figure 4). We attempted to determine whether equine miRNAs show expression patterns representing single transcriptional units. In our analysis, if two miRNAs were within 3 kb of each other, they were considered to be in the same cluster [34]. We found that up to 160 miRNAs were closely co-localized in clusters with the 3 kb threshold. The equine genome contains 51 miRNA clusters with 160 miRNAs accounting for approximately 55% of the known miRNAs (Figure 5). The clusters were variably located on individual chromosome. For example, 40 miRNA genes in 4 clusters were mapped to chromosome 24, while only 2 miRNA genes were mapped to chromosomes 6, 12, 14, 16, 18, and 20 (Table 1).

**Table 1:** Polycistronic miRNAs in horse genome.

Chr	Polycistronic miRNAs	Positions in chr
**2**	miR-200b, miR-200a, miR-429	48455071–48453091
**2**	miR-30e, miR-30c	17435650–17432816
**3**	miR-95, miR-218	103550531–103550551
**3**	miR-328, miR-138	9201088–9201162
**4**	miR-29b, miR-29a	85327107–85326792
**4**	miR-183, miR-96	84472003–84471849
**5**	miR-153-2, miR-101	95527968–95527988
**5**	miR-205, miR-16, miR-15b	15685339–15685252
**6**	miR-141, miR-200c	34423490–34423144
**7**	miR-492-2, miR-708	54764367–54764389
**7**	miR-492-2, miR-24, miR-27a, miR-23a	44930206–44929889
**7**	miR-100, miR-125b	29500017–29500052
**7**	miR-34c, miR-34b	20101717–20101221
**7**	miR-34b, miR-7-2	2932730–2932799
**8**	miR-133a, miR-1	39912260–39912288
**8**	miR-130b, miR-301b	3383110–3382875
**10**	miR-99b, let-7e, miR-125a	21370890–21371640
**11**	miR-497, miR-195	50049193–50048962
**11**	miR-212, miR-132	45640251–45639901
**11**	miR-144, miR-451	42750581–42750479
**12**	miR-194, miR-192	25044429–25044298
**13**	miR-106b, miR-93, miR-25	8034691–8034329
**13**	miR-193b, miR-365-2	30682640–30682716
**14**	miR-143, miR-145	28434698–28433339
**16**	miR-191, let-7g	35442186–35442267
**17**	miR-92a, miR-19b, miR-20a, miR-19a, miR-18a, miR-17	61793120–61792474
**17**	miR-16-2, miR-15a	21138923–21138839
**18**	miR-10b, miR-128-2	19492393–19492419
**20**	miR-582, miR-30c-2	62697786–62697808
**23**	miR-342, let-7f, let-7d	54150817–54148904
**23**	miR-204b-2, miR-7-3	6798843–6798880
**23**	miR-23b, miR-27b, miR-24-2	2317924–2317244
**24**	miR-656, miR-541, miR-496, miR-412, miR-410, miR-409, miR-377	42937011–42930226
	miR-369, miR-154	
**24**	miR-485, miR-134, miR-382, miR-487a, miR-655, miR-544-2, miR-889	42925986-42911169
	miR-539, miR-487b, miR-381, miR-1185, miR-376a, miR-376b, miR-376c	
**24**	miR-495, miR-543, miR-1193, miR-494, miR-329, miR-758, miR-323	42906084–42894709
	miR-1197, miR-380, miR-299, miR-411, miR-379	
**24**	miR-136, miR-432, miR-127, miR-433, miR-431	42748685–42745108
**25**	miR-181b, miR-181a	28673901–28672731
**25**	miR-126, miR-219-2	31667420–31667458
**26**	miR-99a-2, miR-125b-2, let-7c	15508980–15509792
**28**	let-7a, let-7a-2	42016391–42016461
**28**	miR-33a, miR-135a-2	22270420–22270497
**30**	miR-653, miR-181a-2	26398982–26399027
**30**	miR-664, miR-194-2, miR-215	10337974–10337730
**X**	miR-424, miR-503	106954949–106954687
**X**	miR-542, miR-451a, miR-450a, miR-450b	106949741–106948733
**X**	miR-106a, miR-18b, miR-20b, miR-19b-2, miR-92a-2, miR-363	106692701–106691957
**X**	miR-374a, miR-545	55500596–55500517
**X**	miR-374b, miR-421	55435984–55435897
**X**	miR-502, miR-660, miR-500-2, miR-501, miR-362, miR-500	40077407–40073367
**X**	miR-188, miR-532	40068847–40068549
**X**	miR-222, miR-221	37091426–37090756

### Nucleotide bias of equine miRNAs

**Figure 6:**
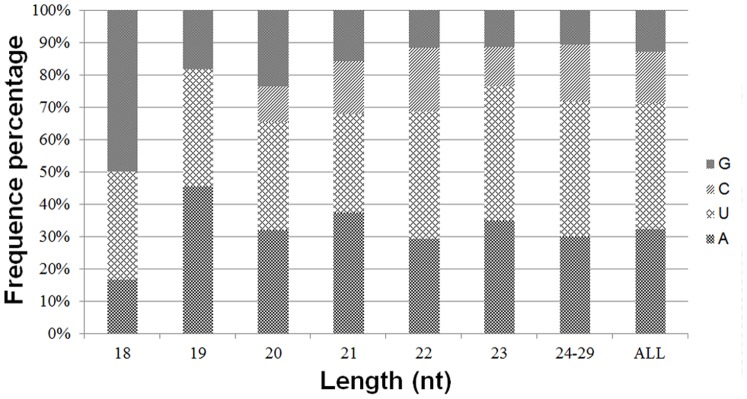
The percentage distribution of the first nucleotide at the 5′ end of all miRNAs of all lengths. Various nucleotides are detected at the 5′ end of miRNA sequences. In general, U is the predominant nucleotide at 5′ end of all miRNAs of all lengths except for miRNAs of lengths 19 and 21.

**Figure 7:**
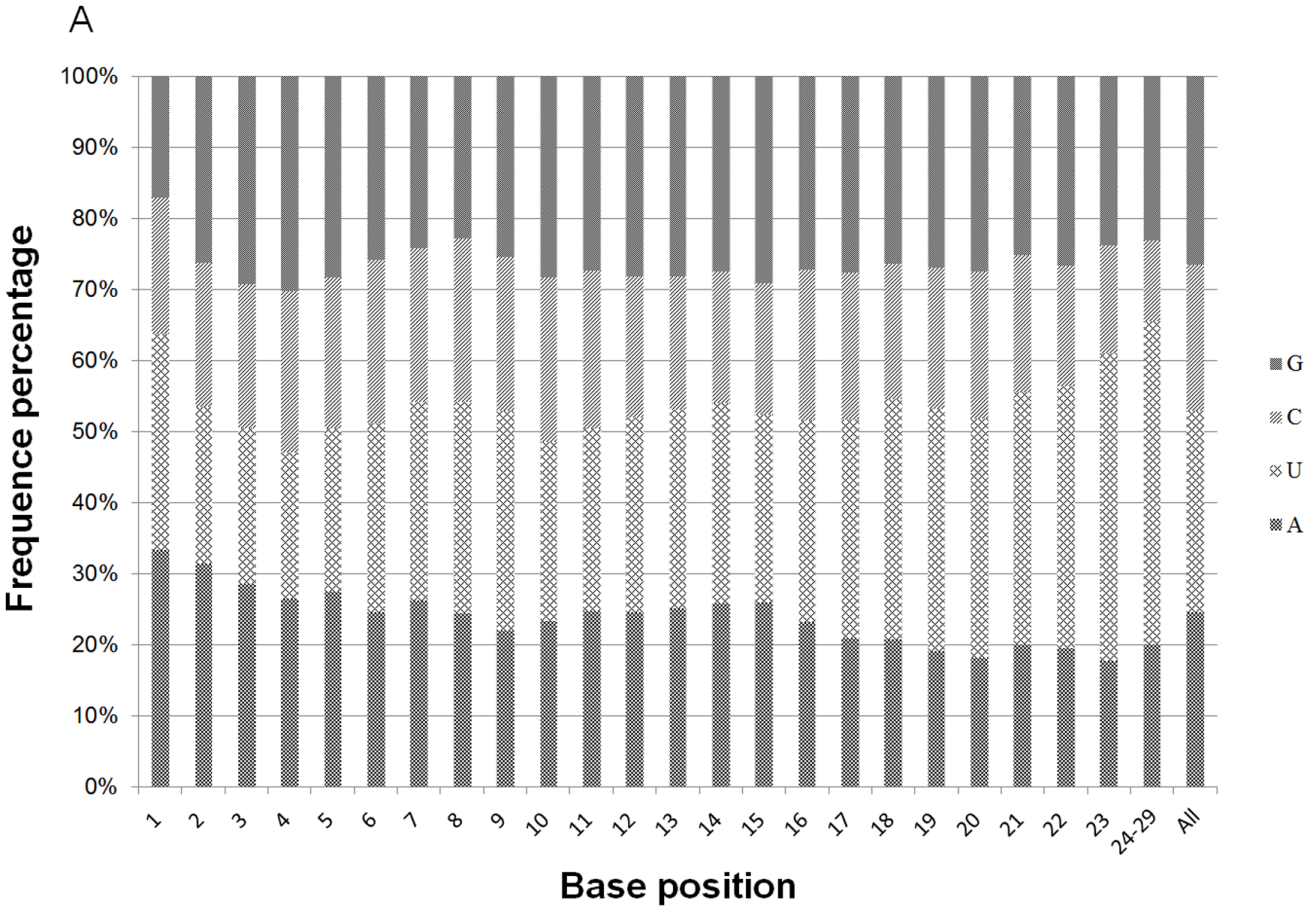
The percentage distribution of base composition at each position of miRNAs. MiRNAs in all combined tissues.

The first nucleotide at the 5′ end of organ-specific miRNAs of any length was predominantly U with a frequency of 40%, 35%, and 43% in muscle, colon, and liver tissues, respectively (Figure 6). Likewise, G and A were the only preferred nucleotide at the 5′ end of 18 and 19 nt long miRNAs in colon, although very short miRNAs that were 18 to 19 nt long were rare in horse. The base composition at each position of all mature miRNAs revealed a clear tendency for U being the most frequently observed nucleotide at specific sites (1, 9, 21, 23–26, and 28). However, C was the least used nucleotide at specific sites (3, 7, 12, 13, 15, 16, 18, 19, 21, 24, and 25) in all tissue miRNA sequences. The distribution of A+U, accounting for an average of 72%, was generally preferred to C+G in all tissue samples (Figure 7). In contrast, from third to sixth nucleotide positions, C+G was more abundant than A+U in all tissue-specific miRNAs. Furthermore, from the second to eighth nucleotide positions, which belong to the seed region of miRNAs, colon miRNAs showed prominent C+G compared to A+U (Data not shown) [35].

## Discussion

Using Illumina short read sequencing technology, a total of 292 known and 329 novel miRNAs were identified in equine tissues including muscle, liver, and colon. Furthermore, it was found that subsets of miRNAs were differentially expressed in horse tissues, suggesting tissue-specific behavior of equine miRNAs. The identification and characterization of equine miRNAs was carried out using NGS technology to obtain millions of small RNA sequences and to construct small RNA differential expression profile in target organs [23]. Although NGS has the advantage of cloning small RNAs exclusively, it has several disadvantages compared to computational approaches [41]. The limitations include the difficulty of finding miRNAs that are expressed at a low level and a cloning inability due to physical properties such as sequence composition and/or post-transcriptional modifications [42]. Further studies are warranted to elucidate a comprehensive list of equine miRNAs that are biologically important.

Although horses are one of the most important domestic animals, research on equine miRNAs has been limited [36]. Attempts have recently been made to investigate the role of equine miRNAs in various conditions. A study using direct sequencing verified two miRNAs, miR-433 and -127, in horse, thus proving the conservation of the miRNAs throughout mammalian species [37]. Zhou et al.[18] discovered a total of 354 mature miRNAs through an integrated computational analysis of an equine genome, providing a baseline for horse miRNA research. Additionally, they presented valuable references for all major sequence characteristics of horse miRNAs, such as the contents of each nucleotide, A+U, C+G, and base composition at each position of pre-miRNA and mature miRNA sequences. Recently, expression profiles of 82 miRNAs were characterized in equine sperms, indicating their role in the regulation of sperm function, fertility, and reproduction [19]. A study revealed that 10 miRNAs were significantly expressed in equine muscles, some of which were also expressed in blood samples [20]. The present study provides the first experimental data on expression profile of global equine miRNAs using NGS technology, some of which were previously reported in an *in silico* study [18].

In horse, the characterization of miRNAs in major organs including liver, skeletal muscle, and large intestine has significant clinical relevance to important equine diseases. Horses are more prone to develop colic, a fatal disease complex resulting from numerous factors including strangulation, obstruction and volvulus [38]. Approximately 34% of horses undergoing an exploratory laparotomy had displacement or volvulus of the large colon due to its free mobility in the abdominal cavity [38]–[39]. Unfortunately, despite the magnitude of the problem of equine colic, relatively little has been known about exact causes. Because of their nature and human usage, horses are affected by various types of muscle diseases including exercise-associated myopathy, post-exhaustion syndrome, and nutritional myopathy [40]. Horses frequently develop liver disease because of their grazing habits, causing significant economic losses [41]. Given that the expression profile of miRNA is specific for organs and/or tissues [3], it is clinically important to discover subsets of organ-specific miRNAs. Our study revealed that both known and novel miRNAs in colon have a larger proportion of total miRNAs than other tissues. In contrast to other mammals, the equine large intestine has relatively complicated anatomical structures and physiological functions to facilitate a steady flow of nutrient [42]. Our data may indicate that the colon-specific miRNAs have more sophisticated roles in the colon's regulatory system [17].

Barrey et al. [20] demonstrated that muscle-specific miRNAs were also detected in blood samples of horses with heritable muscular pathology. The study suggested the potential value of the miRNAs for the development of novel and minimally invasive methodology for diagnosing muscular pathology in horses [20]. Similarly, further studies are warranted to investigate expression profile of tissue-specific miRNAs in the blood and may provide valuable biomarker to identify organ damage and various disease conditions.

Characterization of 5′-end sequences of the miRNA is important because seed sequence of a miRNA is critical in miRNA-target mRNA binding [4]–[43]. Analysis of nucleotide sequences in eukaryotic miRNAs showed a clear bias for U or A at the 5′ position [44]. Our study revealed that U was the most frequent nucleotide at the 5′-end of tissue-specific miRNAs, followed by A. This finding is consistent with results from the published studies on miRNAs of other organisms [18–32–33–45]. In plant, U at the end of 5′ was proposed as being critical for the biogenesis of miRNAs through the recognition of targeted miRNA precursor by RISC [46]. The C+G contents in the 6^th^ position of the seed region were known to induce or enhance the function of a miRNA [45]. In our study, all tissue miRNAs had high percentages of C+G from the third to sixth positions in the sequence. The miRNAs in colon had more distinct sequence bias showing high percentage of C+G in all of the seed nucleotides such a bias may be responsible for distinct functions in colon compared to different organs [45]. The base bias in seed sequence may provide a valuable reference for further study on the identification of regulatory cellular function of miRNAs.

In our study, the equine miRNAs were located on diverse chromosomes. An intriguing feature was that the distribution of miRNAs along the chromosomes was uneven; some chromosomes were relatively miRNA-rich while others were miRNA-poor. Based on chromosomal mapping, more than half of the known miRNAs that exists within 3 kb threshold of each other were considered as a polycistronic transcript. It is interesting to note that our study revealed a number of putative miRNA clusters in horse genome. Consistent with a published study on horse miRNAs [18], a miR-17-92 cluster was also detected on chromosome 17 in our study. This cluster is reportedly harboring miR-17, miR-18a, miR-19a, miR-19b, miR-20a, and miR-92a, and is a well-preserved cluster in human and all vertebrates [11]–[18]. In mammals, the miR-17-92 cluster is divided into two cluster paralogs: miR-106b-25 cluster and the miR-106a-363 cluster [47]. In our study, the former cluster was located on chromosome 13 and the latter cluster on chromosome X. Further studies addressing the precise role of polycistronic transcripts in horse may enhance our understanding about the mechanisms of miRNA generation.

The present study revealed that approximately 53% of the known miRNAs were observed as part of a polycistronic unit. The finding suggests that the clustering property of miRNAs is preserved in horse, although there is partial discrepancy in chromosomal location compared with human data [47]. The proportion of miRNAs in polycistronic units is similar to that of zebrafish miRNAs (50%) [48]. It is noteworthy that approximately 72% of clustered equine miRNAs in our study correspond to those detected by the computational analysis of horse [18]. However, 45 of the 160 miRNAs that are considered as a polycistron could not be identified at orthologous positions in the *in silico* study [18]. We speculated that difference in analysis methodologies is responsible for this discrepancy in miRNA clustering. In our study the miRNA clustering was made based on sequence data from NGS technology, whereas the *in silico* study utilized a limited number of miRNAs identified by BLAST search. Zhou et al. [18] reported polycistronic miRNAs such as miR-302a, -302b, -302c, -302d, and -367 on chromosome 2 and miR-1912 and -1264 on chromosome X, these were not found in our result. In contrast, newly found polycistronic miRNAs in our study were not reported by Zhou et. al [16]. A number of questions for the clustering property for equine miRNAs remain to be clarified in further studies.

## Conclusions

In the present study, we identified 292 known and 329 novel miRNAs in normal equine tissues by using NGS technology. This study significantly enriches the horse miRNA database and presents a valuable reference for equine miRNAs. In addition, the global miRNA expression profile in equine tissues revealed that distinct sets of miRNAs were expressed in a tissue-specific pattern. Clustering in chromosomes and sequence characteristics of miRNA may be useful for dissecting their biological functions in horse. Taken together, the present study suggests that equine miRNAs may play an important role in development and function of specific tissues, and therefore, they can be developed as a valuable molecular marker for various pathophysiological conditions.

## Materials and Methods

### Ethics statement

Animals were sampled with the appropriate consent from horse owners. These horses sacrificed were humanely euthanized following Institutional Animal Care Guidelines for Experimental Animal Use and approved by the Seoul National University Animal Care Committee.

### Tissue collection and high-throughput sequencing of small RNAs

Before the sacrifice, the animals were determined to be healthy based on the results from physical exams, clinical observations and clinicopathologic tests. Major organs including skeletal muscle, liver, and colon were collected immediately after the sacrifice. Collected tissue samples were snap-frozen in liquid nitrogen and stored at −80°C until use. Total RNAs were isolated by a phenol-chloroform method with Trizol reagent (Invitrogen, Carlsbad, CA, USA) according to the manufacturer's recommendations. RNA quality and quantity was determined by Nanodrop (Nanodrop Technologies, Wilmington, DE, USA) and Agilent's 2100 Bioanalyzer (Agilent Technologies, Palo Alto, CA, USA). Total RNAs with high quality were subjected to NGS analysis performed at Theragen Bio Institute (Suwon-city, Gyeonggi-do, The Republic of Korea).

The total RNAs were separated on 15% denaturation polyacrylamide gels, and the band of small RNA fragments between 18 and 32 nt in size were excised. Recovered RNAs from the gels were ligated to a 5′ adaptor and a 3′ adaptor sequentially, and reverse-transcribed to cDNA to obtain sufficient product for Illumina short read sequencing technology (Hiseq 2000). The cDNA products of the small RNA fragments were sequenced directly using Illumina HiSeq 2000 sequencer (Illumina Inc, San Diego, CA, USA) following the Illumina's protocols.

### Sequence analysis of small RNAs

The sequence tags from the NGS were subject to a data cleaning process, which removes the low quality reads, 5′ primer contaminants, reads without 3′ primer or insert tag, reads with poly A, and reads shorter than 18 nt. Then, standard bioinformatics analyses were carried out to annotate the resultant clean tags into different categories. The small RNA tags were annotated using the Rfam RNA family database (http://www.sanger.ac.uk/software/Rfam) and the NCBI GenBank noncoding RNA database (http://www.ncbi.nlm.nih.gov/). Sequences perfectly matching the equine genome along their entire length were subjected to subsequent analyses. Sequences matching known equine small RNAs such as rRNA, scRNA, snoRNA, snRNA and tRNA or degradation fragments of mRNAs were excluded in further analyses.

### Identification of conserved and novel miRNAs

The small RNAs cleaned with Genbank and Rfam were aligned against the miRNA databases, miRBase15.0, to identify the conserved miRNA sequences. Whenever there was miRNA information of horse in miRBase 15.0, perfectly matched sequences were considered as known miRNAs. The small RNAs that cannot be annotated to any category were subjected to novel miRNA prediction software, Mireap (http://sourceforge.net/projects/mireap/), which is utilized to predict novel miRNA by exploring the secondary structure, the Dicer cleavage site and the minimum free energy of the unannotated small RNA tags that could be mapped to the horse genome. Potential novel miRNA candidates should meet the following 10 parameters according to Mireap. (1) minimal miRNA sequence length of 18 nt and maximal miRNA sequence length of 26 nt; (2) minimal miRNA reference sequence length of 20 nt and maximal miRNA reference sequence length of 24 nt; (3) minimal depth of Drosha/Dicer cutting site, 3 nt; (4) maximal copy number of miRNAs on reference, 20 nt; (5) maximal free energy allowed for a miRNA precursor, −18 kcal/mol; (6) maximal space between miRNA and miRNA*, 35 nt; (7) minimal base pairs of miRNA and miRNA*, 14 nt (8) maximal bulge of miRNA and miRNA*, 4 nt; (9) maximal asymmetry of miRNA/miRNA* duplex, 5 nt; and (10) flank sequence length of miRNA precursor, 10 nt. The sequencing data of the conserved and novel miRNAs were deposited in the National Center for Biotechnology Information Sequence Read Archive (http://www.ncbi.nlm.nih.gov/Traces/sra/) under submission number SRA058805.

### Characterization of equine miRNAs

To analyze chromosomal distribution, the known miRNAs identified in the miRBase database were mapped to the genome by an alignment program Short Oligonucleotide Analysis Package software (SOAP, http://soap.genomics.org.cn). The miRNAs on each chromosome were grouped into clusters based on their locations in the equine genome. If two miRNAs were within 3 kb of each other, they were considered as being in the same cluster [34]. Using Illumina Hiseq 2000 sequencer, a more detailed analysis was also performed to further characterize the base composition of miRNA sequences. miRNA Sequences were aligned to the miRNA precursor of horse (mature miRNA if there is no precursor information in miRBase15.0) to predict the base bias in the first position of identified miRNAs of certain length and at each position of all the identified miRNAs.

## Supporting Information

Table S1 **Summary of small RNA sequencing data in muscle, colon, and liver samples.**
(XLSX)

Table S2 **Distribution of the genome-mapped sequence reads in small RNA libraries.**
(XLSX)

Table S3 **Detailed information of all 292 known miRNA genes in horse genome.**
(XLSX)

Table S4 **Detailed information of 166 tissue specific miRNA genes in horse genome.**
(XLSX)

Table S5 **Detailed information of all 329 novel miRNA genes in horse genome.**
(XLSX)

Table S6 **Detailed information of 199 tissue specific novel miRNA genes in horse genome.**
(XLSX)
